# Osteoregeneration of Critical-Size Defects Using Hydroxyapatite–Chitosan and Silver–Chitosan Nanocomposites

**DOI:** 10.3390/nano13020321

**Published:** 2023-01-12

**Authors:** Miguel A. Casillas-Santana, Yael N. Slavin, Peng Zhang, Nereyda Niño-Martínez, Horacio Bach, Gabriel A. Martínez-Castañón

**Affiliations:** 1 Laboratorio de Nanobiomateriales, Facultad de Estomatología, Universidad Autónoma de San Luis Potosí, San Luis Potosí 78290, Mexico; 2 Division of Infectious Diseases, Faculty of Medicine, University of British Columbia, Vancouver, BC V6G 3Z6, Canada; 3 Facultad de Ciencias, Universidad Autónoma de San Luis Potosi, San Luis Potosí 78295, Mexico

**Keywords:** silver nanoparticles, hydroxyapatite particles, chitosan, critical-size defect, animal model

## Abstract

Bone is a natural nanocomposite composed of proteins and minerals that can regenerate itself. However, there are conditions in which this process is impaired, such as extensive bone defects and infections of the bone or surrounding tissue. This study evaluates the osteoregenerative capacity of bone grafting materials in animals with induced bone defects. Colloidal chitosan dispersion nanocomposites, nanohydroxyapatite–chitosan (NHAP-Q) and nanosilver–chitosan (AgNP-Q), were synthesized and characterized. Non-critical-size defects in Wistar rats were used to evaluate the material’s biocompatibility, and critical-size defects in the calvarias of guinea pigs were used to evaluate the regenerative capacity of the bones. Moreover, the toxicity of the nanocomposites was evaluated in the heart, liver, spleen, kidneys, and skin. Histological, radiographic, and electron microscopy tests were also performed. The results showed that neither material produced pathological changes. Radiographic examination showed a significant reduction in defects (75.1% for NHAP-Q and 79.3% for AgNP-Q), angiogenesis, and trabecular formation. A toxicological assessment of all the organs did not show changes in the ultrastructure of tissues, and the distribution of silver was different for different organs (spleen > skin > heart > kidney > liver). The results suggest that both materials are highly biocompatible, and AgNP-Q achieved similar bone regeneration to that reported with autologous bone. The main research outcome of the present study was the combination of two types of NPs to enhance antimicrobial and osteoregeneration activities. These colloidal chitosan dispersions show promise as future biomaterials in the medical field for applications in fast-healing fractures, including broken bones in the oral cavity and hip replacement infections.

## 1. Introduction

Bone is a natural nanocomposite composed of proteins and minerals, which has the mechanisms and the intrinsic capacity to regenerate by itself [1]. However, there are conditions in which this process is impaired, such as extensive bone defects and bone or surrounding tissue infections. Furthermore, bone tissue may be lost due to trauma or periodontal disease, representing a challenge for orthopedists, maxillofacial surgeons, and reconstructive specialists. 

In adults, only minor bone defects can heal spontaneously [2]. The deterioration in wound repair is related to the rapid internal growth of soft tissue towards the bone defect, thus inhibiting bone proliferation and formation at the edges of the bone defect [3]. One of the significant challenges oral and maxillofacial surgeons face is the restoration of defects in the maxillofacial bones. 

Autogenous bone grafts are the preferred material when craniofacial reconstruction is required [4]. Although highly effective, many problems are associated with their use. For example, autogenous grafts can increase the operative time by increasing the risks of infection, donor site pain, and morbidity [5,6], and they are often mechanically unstable during surgery and might be insufficient for reconstructing extensive defects. These grafts may also undergo resorption, requiring subsequent surgical procedures. As a result, there has been broad interest in generating biomaterials that provide optimum conditions for cells with the osteogenic potential to minimize these problems and angiogenesis.

Hydroxyapatite (HA) is a calcium phosphate ceramic material that is the main mineral component of vertebrates. Depending on its source, it may have a structure similar to bone tissue. HA possesses the characteristic of osteoconductivity, allowing the connective tissue to penetrate the surrounding bone, inducing a process of ossification. In addition, calcium phosphates can bind and concentrate endogenous bone morphogenic proteins, leading to osteoinduction (cell recruitment and stimulation leading to pre-osteoblast development) [7]. Combining HA with chitosan (deacetylated chitin) improves the poor mechanical properties of HA, making it a promising material for biomedical applications due to its biocompatibility. It also improves its ability to facilitate the regenerative process in wounds. For example, chitosan combined with nano-HA in the form of spheres and implanted in calvarial (skullcap) defects promoted bone regeneration in 12 weeks [8,9]. Chitosan alone has also been shown to enhance cellular activity, inducing proliferation (reaching a plateau at 0.1 mg/mL) in human periodontal ligament fibroblasts, as well as upregulating alkaline phosphatase (ALP) activity (known to be a marker of osteogenic differentiation) and increasing type I collagen production [10]. 

Silver nanoparticles (AgNPs) have been widely used for their bactericidal effect and their ability to reduce bacterial skin infections [11,12,13]. Moreover, it was reported that AgNPs promoted regeneration tissue in a rat model of thermal damage to the back [14]. However, in this study, the partial-thickness wound typically healed after approximately 35 days. Still, in animals treated topically with AgNPs, the wounds healed in 26 days, indicating that AgNPs could modulate the local and systemic inflammatory response after burn injury by modulating the cytokine profile [14].

Studies have shown that colloidal chitosan dispersions have a high potential to mimic the extracellular matrix [15,16,17]. This is because cells and tissues are immersed within a 3D lattice in vivo, constituting a complex extracellular environment with high porosity. In contrast, a 2D culture system is too simple to parameterize the native environment [18,19].

This study aimed to evaluate the biocompatibility of colloidal nano-HA-chitosan (NHAP-Q) and AgNP-chitosan (AgNP-Q) dispersions and their capacity to improve bone repair processes in critical-size defects using animal models. This study is novel because it showed antibacterial and osteoregenerative activities when a colloidal chitosan dispersion was prepared with AgNPs and NHAPs. 

## 2. Methods

### 2.1. Synthesis of NPs

AgNPs were synthesized by dissolving 0.169 g of AgNO_3_ (Fermont, Monterrey, Mexico) in 100 mL of deionized water. Then, 0.1 g of gallic acid (Sigma-Aldrich, St. Louis, MO, USA) previously dissolved in 10 mL of deionized water was added, and the pH was immediately adjusted to 11 using 1.0 M NaOH solution. This dispersion was kept in the dark at room temperature. 

The synthesis of NHAP was carried out in an aqueous solution using a precipitation technique. First, a reaction was prepared by briefly mixing 50 mL of a 1 M aqueous solution of Ca(NO_3_)_2_·4H_2_O with 50 mL of 0.6 M KH_2_PO_4_, adjusting the pH to 10 with dropwise addition of NH_4_OH. The reaction was kept under magnetic stirring for 1 h and allowed to settle for 24 h. The next day, the formed nanoparticles were washed with deionized water (×4) and then heated at 900 °C for 1 h. The powders were kept at room temperature.

### 2.2. Colloidal Chitosan Dispersion Synthesis

The AgNP-Q and NHAP-Q colloidal chitosan dispersions were prepared with a minor modification of published protocols [20]. Briefly, 2 mL of acetic acid was mixed with 48 mL of the AgNP or NHAP solutions, after which 1.95 g of chitosan (310–375 kDa, Sigma-Aldrich) and 0.2 mL of glycerol were added under magnetic stirring until the gel was formed.

### 2.3. Characterization of the NPs

The NPs used in this study were characterized by transmission electron microscopy (TEM, JEOL, JEM-1230, Tokyo, Japan) operated at 100 kV. In addition, dynamic light scattering (DLS) was used to measure the size distribution of the particles, and their zeta potential was measured using a Zetasizer Nano ZS (Malvern Instruments, Malvern, Worcestershire, United Kingdom) operating at 25 °C with a He-Ne laser at a wavelength of 633 nm and a detection angle of 90°.

To confirm the composition of NPs, X-ray diffraction analysis was performed using dried powders of the NPs. X-ray diffraction patterns were recorded on a GBC-Difftech MMA model, GBC, Braeside, Victoria, Australia), with Cu Kα irradiation at λ = 1.54 Å. In addition, the Vis-NIR absorption spectra of AgNPs were obtained using a CHEMUSB4-VIS-NIR (Ocean Optics, Dunedin, FL, USA) spectrophotometer.

### 2.4. Rheological Characterization of the Colloidal Chitosan Dispersions

The colloidal chitosan dispersions were characterized using a rheometry test (RT) and environmental scanning electron microscopy (ESEM). The rheometry test was performed with a U.S. 200 Paar Physica Rheometer equipped with Peltier temperature control. The determination was carried out using a cone–plate geometry (MK31, 50 mm in diameter, 1° tilt, and 0.49-micron truncation). An aliquot of the sample was deposited at the base of the plate to secure it further. The distribution of the NPs in the gels was observed using an environmental scanning electron microscope (ESEM Quanta 200, FEI, Hillsboro, OR, USA), and no special sample preparation was needed for this observation.

### 2.5. Bactericidal Activity

A panel of four multidrug-resistant pathogenic bacteria was used to determine the antibacterial activity of the colloidal chitosan dispersions. Bacterial strains clinically isolated from the Hospital Central Dr. Ignacio Morones Prieto (San Luis Potosí, SLP, México) were tested and identified as previously described [21]. Strains tested in this study included the Gram-positive *Staphylococcus aureus* 700 (resistance: ciprofloxacin, clindamycin, erythromycin, oxacillin, and penicillin), *Staphylococcus epidermidis* 710 (resistance: penicillin, oxacillin, gentamicin), and *Staphylococcus epidermidis* 6 (resistance: penicillin, erythromycin), and the Gram-negative *Acinetobacter baumannii* 808 (resistance: meropenem). Strains were cultured in Mueller–Hinton broth (BD) and were maintained on solidified broth with 1.5% agar at 4 °C. Bacterial strains were exposed to concentrations of 2.5 to 25 μg/mL. The minimal inhibitory concentration (MIC) was measured following published protocols [22], defined as the concentration at which no bacterial growth was observed.

### 2.6. Cytotoxic Activity of Osteoblasts and Macrophages

Primary osteoblast cells were obtained from mice in the in vivo study (see below). First, mouse femurs were processed according to published protocols [23]. Briefly, femurs were scraped with a scalpel (#11) to clean the remainder of the flesh, and the bone marrow was flushed with PBS using a syringe (5 mL) and a needle (27G). Next, femurs were cut into 1–2 cm pieces using sterile scissors and washed in PBS (×4) for 5 min each. Then, 4 mL of a solution of collagenase II (Sigma-Aldrich) was added, and the bones were vigorously shaken at 37 °C for 2 h to remove any debris. Next, the bone pieces were transferred to tissue culture dishes (20 pieces/dish) and cultured with a medium containing DMEM (Sigma-Aldrich) supplemented with antibiotics (100 U/mL penicillin, 50 μg/mL streptomycin, and 50 μg/mL gentamycin, Invitrogen, Waltham, MA, USA), amphotericin B (1.25 μg/mL, Fungizone), 100 μg/mL ascorbate (Sigma-Aldrich), and 10% fetal calf serum (Invitrogen). 

An alkaline phosphatase (ALP) test [24] was performed to confirm the identity of the osteoblast: 3 × 10^5^ cells/well were dispensed on a 96-well plate overnight at 37 °C supplemented with 5% CO_2_. The next day, cells were carefully washed with PBS (×2) and fixed with 10% formalin for 1 min. Then, the formalin solution was aspirated, and cells were washed with DMEM supplemented with 0.05% Tween-20. The staining of the cells was completed after the addition of the substrate 5-bromo-4-chloro-3-indolyl-phosphate (BCIP) (175 μg/mL from a stock of 50 mg/mL in 70% dimethylformamide (DMF)) and 4-nitro blue tetrazolium chloride (NBT) (225 μg/mL from a stock solution of 50 mg/mL in 70% dimethyl formamide). Finally, cells were incubated at room temperature in the dark for ~10 min and analyzed under a microscope.

All the experiments involving cells were cultured at 37 °C in an atmosphere supplemented with 5% CO_2_. The human-derived monocyte cell line THP-1 (ATCC TIB-202) was used in this study to measure the cytotoxic effects of the colloidal chitosan dispersions. Cells were grown in tissue culture flasks using RPMI 1640 (Sigma-Aldrich), supplemented with 10% fetal calf serum (FCS) (Invitrogen), 2 mM L-glutamine, and amphotericin B (2 μg/mL, Fungizone). THP-1 cells (3 × 10^5^/well in a 96-well plate) became adherent upon supplementation of 40 ng/mL phorbol 12-myristate 13-acetate (PMA, Sigma-Aldrich). 

Osteoblasts and macrophages were exposed to the colloidal chitosan dispersions at the following concentrations: 1.25, 2.5, 5, 10, and 20 μg/mL for 18 h. Each treatment was performed in triplicate. Untreated cells were used as a negative control, whereas cells treated with 0.1% Tween-20 (Fisher) were used as a positive control. 

The cytotoxicity was measured using the 3-(4,5-dimethylthiazol-2-yl)-2,5-diphenyltetrazolium bromide (MTT) assay as published previously [25].

### 2.7. Osteoblast Proliferation Assay

To assess osteoblast proliferation, the 5-bromo-2′-deoxyuridine (BrdU) assay was used (BrdU Cell Proliferation Assay Kit, Cell Signaling Technologies, Danvers, MA, USA) according to the manufacturer’s instructions. Briefly, osteoblast cells were cultured as described above, and 2 × 10^4^ cells were dispensed in a 96-well plate. Once the cells adhered to the bottom of the well, a BrdU solution was added, and the plates were incubated at 37 °C supplemented with 5% CO_2_ for 48 h. At 24 h, the medium of one plate was carefully disposed of, and 100 µL/well of the fixing/denaturing solution was added. The plate was left at room temperature for 30 min, and 100 µL/well of the detection antibody solution was added after removing the previous solution. After 1 h incubation at room temperature, the solution was removed, and the plate was washed (×3) with the wash buffer. Then, 100 µL/well of HRP-conjugated secondary antibody solution was added, and the plate was kept at room temperature for 30 min. Lastly, the wells were rewashed with the wash buffer (×3). To develop the test, 100 µL of the TMB substrate was added and incubated for 30 min at room temperature. To finalize the reaction, 100 µL of the stop solution was added, and the absorbance at 450 nm was measured using a plate reader (Epoch, Biotek, Winooski, VT, USA).

### 2.8. Anti-Inflammatory Assay

THP-1 cells at a concentration of 5 × 10^4^ cells/well were dispensed on a 96-well plate and activated after adding 100 ng/mL PMA (Sigma-Aldrich). Plates were incubated at 37 °C in a humidified atmosphere of 5% CO_2_ for 24 h. Then, adherent cells were gently washed with fresh medium (×3) and incubated in the presence of the compounds for 6 h. After removing the compounds and washing the cells, an immunological response was initiated after adding 500 ng/mL of *E. coli* lipopolysaccharide (LPS) (Sigma-Aldrich). After 12 h, supernatants were transferred to a new 96-well plate and kept at −20 °C for further analysis. The pro-inflammatory cytokines interleukin-6 (IL-6) and tumor necrosis factor-alpha (TNF-α) and the anti-inflammatory interleukin-10 (IL-10) were used to evaluate the inflammatory response using a kit (R&D) and according to the instructions of the manufacturer. DMSO alone and DMSO combined with LPS were used as negative controls, whereas prednisone (1 mM, Sigma-Aldrich) was used as a positive control. 

### 2.9. Animal Models

This study was evaluated and approved by the ethics committee of the U.A.S.L.P. under the protocol CIS-FE-028-014. Furthermore, the experiments were conducted following the Mexican Legislation Standard of NOM-063-SSA1-1993 and the General Health Law (Guide for the Care and Use of Laboratory Animals, The National Academies Press).

Three protocols were used in this study: Protocol A evaluated the biocompatibility of the materials. In this protocol, three defects of non-critical size were performed on the tibial axis of the limb in Wistar rats (*n* = 5), weighing between 400–500 g, and performed in duplicate. Colloidal NHAP-Q chitosan dispersion was implanted in defect 1 (Group 1), colloidal AgNP-Q chitosan dispersion in defect 2 (Group 2), and a blood clot in defect 3 as a control group (Group 3). Protocol B evaluated the regenerative capacity of the materials. In this protocol, two critical defects (8 mm each) were made on the calvaria of guinea pigs (*n* = 10) weighing between 700 and 900 g. Colloidal NHAP-Q chitosan dispersion was implanted in the right defect (Group 1), and colloidal AgNP-Q chitosan dispersion was implanted in the left defect (Group 2). Protocol C was used to measure the distribution of Ag in different organs. In this protocol, colloidal AgNP-Q chitosan dispersions were sub-dermally implanted in the dorsal region of guinea pigs (*n* = 5). Another group of guinea pigs was sub-dermally injected with saline as a control. 

According to the experimental protocol, the animals were randomly assigned to two or three groups with food and water access ad libitum.

### 2.10. Surgical Procedure

In protocol A, all rats were anesthetized with an intraperitoneal injection of ketamine (33 mg/kg, Anesket, ketamine hydrochloride 1000 mg/10 mL). Then, the right limb was shaved, and the skin surface was disinfected with iodopovidone solution (DermoDine Iodopovidone 11 g/100 mL). Before surgery, the bone was exposed through an incision approximately 2 cm in length using a scalpel blade (#15). Three defects with a diameter of 2 mm were made on the tibial axis of the right limb using a carbide burr # 3 under constant irrigation with saline solution to prevent overheating. The first defect was implanted with colloidal NHAP-Q chitosan dispersion to fill the cavity evenly, the second defect was implanted with colloidal AgNP-Q chitosan dispersion, and the third defect was implanted with a blood clot to be used as a control (Figure S1). After 2 weeks, the same procedure was performed on the animal’s left limb. After an evolution period of 2 weeks, the right limb had a repair process of 4 weeks (Figure S2). 

In protocol B, guinea pigs were sedated with an intraperitoneal injection of ketamine (40 mg/kg, Anesket, ketamine hydrochloride 1000 mg/10 mL). Then, the parietal–frontal skin was disinfected with iodopovidone (DermoDine). Before surgery, an incision of approximately 3 cm was made with a scalpel blade (#15) from the nasofrontal suture to the external occipital protuberance to expose the parietal bone after raising the flaps with a periosteal elevator. Two defects of critical size (8 mm diameter each) were made in the calvaria using a carbide cutter (#8) at low speed (maximum speed of 500 rpm) with continuous and copious irrigation with saline solution to avoid overheating. Special care was taken to prevent damage to the dura mater, and the diameter of the defects was confirmed with a periodontal probe. The defect in the right parietal was implanted with a colloidal NHAP-Q chitosan dispersion and the left parietal defect with a colloidal AgNP-Q chitosan dispersion. Guinea pigs were sacrificed at 4 or 8 weeks, and the calvarias were removed with a diamond disk (600 grit) at low speed.

In protocol C, the guinea pigs were sedated with ether. After complete disinfection of the interscapular zone (between the shoulder blades) with iodopovidone (DermoDine), two incisions (left and right) of approximately 2 cm were performed, and 100 mg of colloidal AgNP-Q chitosan dispersion was sub-dermally injected in the left dorsal incision. In contrast, the right incision was maintained as control. Animals were sutured and euthanized after a postoperative period of 5 weeks. The targeted organs, heart, liver, spleen, kidneys, and the skin surrounding the implantation zone, were extracted.

In all the protocols mentioned above, once the materials were implanted, the open wounds were sutured with black silk 4-0. The animals were recovered with light and dark cycles of 12/12 h and the antibiotic ceftriaxone (140 mg/kg) and the analgesic metamizole (570 mg/kg) for 3 d. Weight fluctuations were monitored daily.

### 2.11. Histological and Radiographic Analyses

Samples obtained in all the protocols above were stored in 10% formaldehyde and refrigerated at 4 °C. For protocols A and B, the pieces were decalcified with 10% HNO_3_ under constant agitation for 72 h and 7 days, respectively, and histological staining was performed with hematoxylin-eosin. For protocols A and B, three random sections of each defect were taken for histological examination. The de novo bone formation, residual material, pathological changes, and inflammatory response were investigated in each section. In protocol C, ultrastructural changes and the presence of inflammatory filtrates were examined by a pathologist under a light microscope with a magnification of 10×, 20×, and 40×. In addition, each image was digitally captured with a camera attached to the microscope for further analysis.

For protocol B, before histological processing, a conventional X-ray of the calvaria was taken with an angle of 90°, a distance of 10 cm, and an exposure of 14 milliseconds using a 5 MP Samsung camera (2592 × 1944 pixels, autofocus, LED flash). Then, radiographic analysis was performed with the program ImageJ. 

### 2.12. Blood Parameters

Blood samples were collected by a direct puncture in the auricle or descending aorta in the heart after sedation with ether. A sample of 2 mL of blood was drained per guinea pig and divided into two portions. The first portion (250 μL) was collected in a Vacutainer supplemented with EDTA, and the rest of the sample was placed in a centrifuge tube. 

A complete blood count was performed, and the number of lymphocytes (LYM), neutrophils (GRAN), erythrocytes (RBC), hemoglobin (HGB), hematocrit (HCT), mean corpuscular volume (MCV), mean corpuscular hemoglobin (MCH), mean corpuscular hemoglobin concentration (MCHC), red blood cell distribution width (RDW), platelets (PLT), and mean platelet volume (MPV) were measured using a blood cell counter (Abbott Diagnostics Cell-Dyn 1700 and Alcyon 300, Ramsey, Minnesota, USA). In addition, the renal and hepatic functions were assessed by measuring AST, ALT, urea, and creatinine using a biochemical blood analyzer (Biosystems A15, Barcelona, Spain). The assays followed international standards for the clinic and hematology [26]. 

### 2.13. Ag Content in Tissues

The heart, kidney, liver, skin, and spleen were collected and analyzed for their Ag content (*n* = 5). Each organ (1 g) was homogenized and digested with an aqueous solution of HNO_3_ (32%, 10 mL) for 48 h at 50 °C. After complete disintegration, 1 mL of the organ digestion was mixed with 9 mL of deionized water, and the Ag analysis was performed by atomic absorption (AA-7000, Shimadzu, Kyoto, Japan). 

### 2.14. Histopathology Analysis of the Defects

Samples were stored in formaldehyde (10%) and kept at 4 °C. The samples were decalcified with HNO_3_ (10%) under constant agitation for 72 h (protocol A) or 7 d (protocol B). The histological analysis was performed in three random sections after staining with hematoxylin–eosin. For protocols A and B, each section was analyzed for de novo ossification, pathological changes, and the presence of immune cells. In protocol C, ultrastructural changes and white cell infiltration were assessed. The analyses were performed by a pathologist, who was not exposed to the identity of each sample. 

### 2.15. Analysis of Calvarias

For protocol B, guinea pig calvarias were dissected and removed at the end of the 8 weeks and prepared conventionally for histology but were not stained. Histological sections were placed on the object holders without the protection of the resin; they were covered with gold (fine-coat ion sputter JFC 1100) and taken to a scanning electron microscope (JEOL, JSM-6610) for histomorphometric interpretation at 10 kV, at 10 mm of working distance, and using a secondary electron detector.

The guinea pig calvarias were removed and prepared conventionally for their histological analysis. However, they were not stained and covered with resin. Instead, they were covered with gold before the last step for their proper observation. For this observation, at least five random points of each calvaria were assessed.

### 2.16. Statistical Analysis

A student *t*-test was used for statistical analysis. A *p*-value < 0.05 was considered significant. GraphPad Prism version 6.00 for Mac (San Diego, CA, USA) was used for the statistical calculations.

## 3. Results

### 3.1. NP Characterization

**Figure 1:**
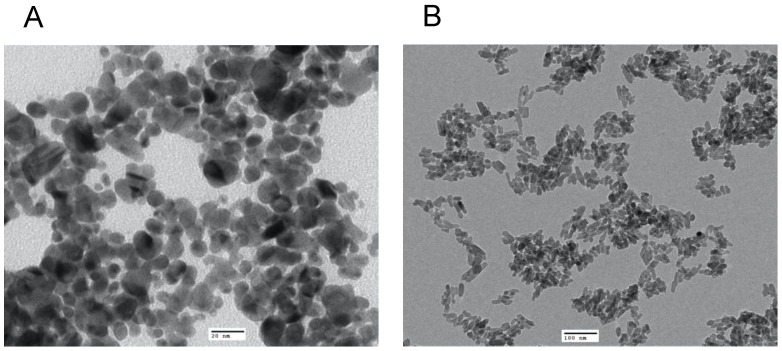
Characterization of the NPs used in this study by TEM analysis of (**A**) AgNPs, scale bar: 20 nm and (**B**) HANPs, scale bar: 100 nm.

TEM revealed that the average size of the AgNP was 13 nm with a spherical shape (Figure 1A), whereas the NHAP ranged from 19 to 32 nm with a rod-like shape (Figure 1B). The NHAP synthesized in this study resembled the bone matrix of 70% crystalline HA, typically measured at 20–80 nm in length and 2–5 nm in thickness [27]. The zeta potential results were −48.4 and −26.8 mV for the AgNPs and NHAP, respectively (Figure S3). Based on the characterization results, both NPs will not agglomerate because of the high negative zeta potential. 

**Figure 2:**
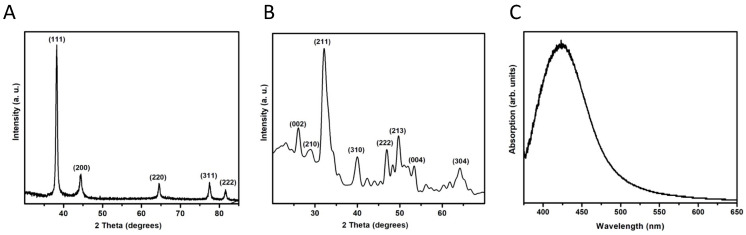
X-ray diffraction patterns of the NPs synthesized in this study. (**A**) AgNPs. (**B**) NHAPs. (**C**) Surface plasmon resonance of AgNPs.

The AgNPs were identified as elemental Ag after comparing the diffraction pattern with the published data (JCPDS card no. 04-0783). The three most intense peaks correspond to the planes (111), (200), and (220) (Figure 2A). These results indicate that there is no preferential growth of the crystals. In the case of the NHAP, it can be indexed as hexagonal HA compared with JCPDS card no. 09-0432; no other phases or compounds were detected, indicating that the prepared samples were pure NHAP (Figure 2B). By observing the broadening of the diffraction peaks, it seems that AgNPs present excellent crystallinity, whereas NHAPs are slightly less crystalline.

### 3.2. Rheological Characterization of the Colloidal Chitosan Dispersions

**Figure 3:**
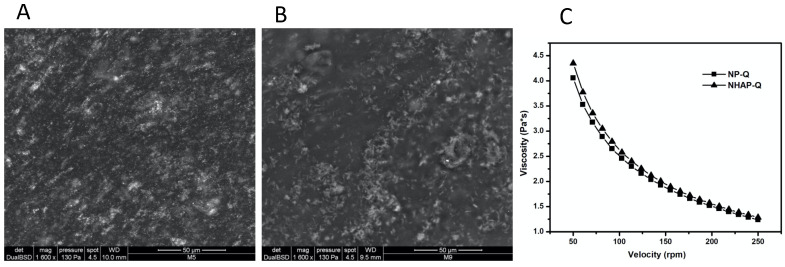
Rheological characterization of the colloidal chitosan dispersions. ESEM was used to characterize the (**A**) AgNP-Q (scale bar: 50 μm) and (**B**) colloidal NHAP-Q chitosan dispersions (scale bar: 50 μm). (**C**) The viscosity of both colloidal chitosan dispersions was measured.

ESEM images showed that once AgNPs and NHAP agglomerated, they were incorporated into the matrix (Figure 3). However, ESEM images showed good distribution of these NPs in the gel matrix. In addition, the colloidal chitosan dispersions showed a pseudoplastic or shear-thinning behavior (viscosity decreases when the velocity increases). This property could be used when colloidal chitosan dispersions are used. This would be advantageous since the dispersion would become more fluid while it is manipulated, leading to an easier distribution on the surface. In addition, the viscosity would probably increase when the stress ceased, preventing the dispersion from flowing from the application site.

### 3.3. Bactericidal Activity of the Colloidal Chitosan Dispersions

A potent antibacterial activity was measured when the colloidal chitosan dispersions were tested against four Gram-positive and Gram-negative multidrug-resistant bacterial strains (Table 1). A range of MICs between 7.5 and 20 μg/mL was measured.

### 3.4. Cytotoxicity and Proliferation Assays

**Figure 4:**
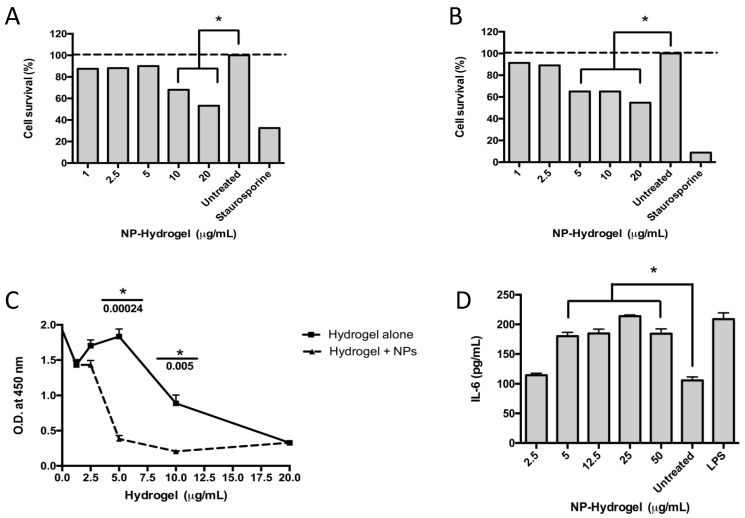
Effect of the colloidal chitosan dispersions on cytotoxicity and the immune response. Macrophages (**A**) and osteoblasts (**B**) were exposed to different concentrations of colloidal AgNP-Q chitosan dispersions, and the cytotoxicity was measured using the MTT assay and according to the experimental methodology. Staurosporine was used as a positive control, whereas untreated cells were used as a negative control. (**C**) A proliferation assay was performed in osteoblasts using the BrdU test over 48 h and according to the methodology. (**D**) An immunological response was measured using the pro-inflammatory cytokine IL-6 measured in the supernatant of the culture. LPS (lipopolysaccharide) and untreated cells were used as positive and negative controls, respectively. Colloidal NP-chitosan dispersion, colloidal AgNP-Q chitosan dispersion. All the experiments were performed in triplicate. The asterisk represents a *p*-value of <0.05. Data shown are the mean ± S.D.

The cytotoxic effects of the colloidal NHAP-Q/AgNP-Q chitosan dispersions on primary osteoblasts and the human monocyte cell line THP-1 were assessed. The results showed toxicity at 5 and 12.5 μg/mL when incubated with osteoblasts and macrophages, respectively (Figure 4A,B).

Different concentrations of the colloidal NHAP-Q and AgNP-Q chitosan dispersions were exposed to osteoblasts to determine whether the colloidal chitosan dispersions were detrimental to cellular proliferation. The colloidal chitosan dispersion lacking the AgNPs was used as a negative control. The results showed that the colloidal NHAP-Q and AgNP-Q chitosan dispersions did not affect the proliferation of the cells at concentrations of <5 μg/mL as measured by BrdU incorporation into the DNA (Figure 4C). However, at concentrations of >5 μg/mL, a significant decrease in DNA incorporation was measured compared with the negative control, suggesting inhibition of osteoblast growth. Interestingly, at concentrations of 20 μg/mL, both colloidal NHAP-Q and AgNP-Q chitosan dispersions and the colloidal chitosan dispersion inhibited osteoblast proliferation. 

### 3.5. Immunological Response

To determine whether the exposure of macrophages to the colloidal chitosan dispersions would modulate an immunological response, the levels of the pro-inflammatory cytokines IL-6 and TNF-α and the anti-inflammatory IL-10 cytokine were measured. A significant increase of ~60% in the concentration of IL-6 was measured compared with the untreated control. This increase was observed when macrophages were exposed to AgNP- colloidal chitosan dispersion of ≥5 μg/mL (Figure 4D). Interestingly, TNF-α and IL-10 were not significantly changed when macrophages were exposed to colloidal NP-chitosan dispersion concentrations ranging between 5 and 50 μg/mL. 

### 3.6. Postoperative Assessment

The repair process progressed in protocols A and B without any sign of infection. No changes in animal behavior, paralysis, shortness of breath, or signs of pain were recorded. Furthermore, the weight curves were normal in all animals with a progressive increase in weight, which indirectly reflected that neither the grafted material, colloidal NHAP-Q or AgNP-Q chitosan dispersions, nor the surgical procedure produced any changes in the animal that modified its behavior for food and drink intake.

### 3.7. Histological Analysis

**Figure 5:**
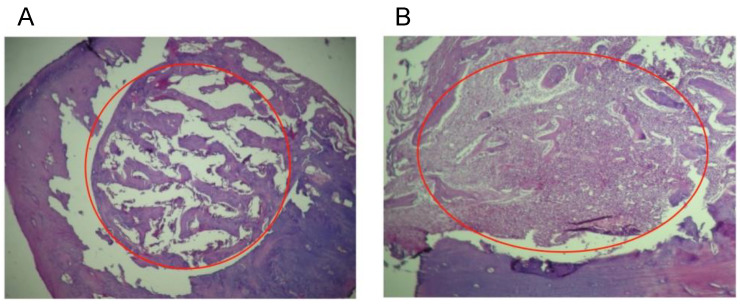
Histological analyses of the defect implanted in the animals. (**A**) The defect was implanted with colloidal NHAP-Q chitosan dispersion (protocol A, Group 1, 4 weeks). (**B**) The defect was implanted with colloidal AgNP-Q chitosan dispersion (protocol A, Group 2, 4 weeks). No signs of significant inflammation were observed in both images. Magnification, 10×. Hematoxylin and eosin were used for staining. The red circle corresponds to the area of the defect.

For a qualitative evaluation of the inflammatory response in the tissues, the levels of response observed were defined as: (1) when the grafts do not stimulate any inflammatory response; (2) when there is a presence of macrophages and plasma cells, which means that the material has been detected in the local tissue and presented to B lymphocytes; (3) a moderate immune response with the presence of giant cells; and (4) a severe inflammatory response accompanied by necrosis [20]. In our study, the histological evaluation in protocol A (biocompatibility) showed that the repair process was normal with no changes such as pro-inflammatory cells, bone resorption, any degree of inflammation, or necrosis. However, for Group 1 (colloidal NHAP-Q chitosan dispersion), the histological results revealed a slight bone growth on the margins toward the central part of the defect, presumably because the implanted material was uniformly distributed throughout the defect. Still, some particles remained present after 2 weeks and 4 weeks with few signs of resorption (Figure 5A).

For Group 2 (colloidal AgNP-Q chitosan dispersion), the histological observation showed small amounts of residual material at 2 weeks with improved resorption of the implanted material at 4 weeks, as well as the formation of trabecular bone very similar to basal bone (Figure 5B). It is important to mention that none of the materials tested in this study stimulated the implanted materials’ pro-inflammatory or rejection process. On the contrary, it is precisely the formation of new bone material which confirms that both materials are biocompatible, highlighting the biodegradation rate of the chitosan and the level of purification of AgNPs in the colloidal AgNP-Q chitosan dispersion.

On the other hand, in protocol B, whose main objective was to evaluate the regenerative capacity of the materials in a critical-size defect, histological sections of the calvarias were assessed at 4 weeks after implantation of the defects with colloidal NHAP-Q chitosan dispersion. Histological examination showed the formation of bone trabeculae, especially in the central part of the defect and some areas near the edges, and connective tissue with fewer osteoblasts and osteocytes. The presence of connective tissue suggests the presence of residual material that prevents correct repair of the bone with some signs of degradation. 

**Figure 6:**
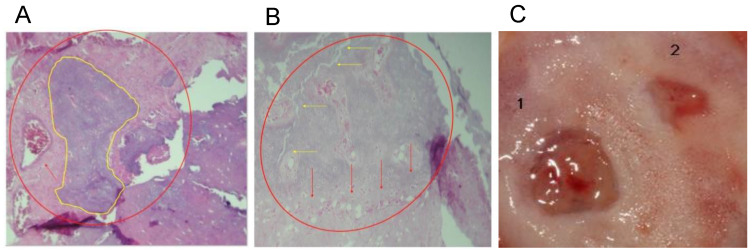
Histological analyses of the defect implanted in the animals. (**A**) The defect was implanted with colloidal NHAP-Q chitosan dispersion (protocol B, Group 1, 4 weeks). Mineralized tissue is shown in yellow, and the red arrow points to connective tissue. (**B**) The defect was implanted with colloidal NHAP-Q chitosan dispersion (protocol B, Group 1, 8 weeks). Red arrows point to the formation of laminar bone, and yellow arrows point to the bony bridge between the new and basal bone. (**C**) Clinical view at 8 weeks using (1) NHAP-Q and (2) colloidal AgNP-Q chitosan dispersion. Magnification, 10×. Hematoxylin and eosin.

After 8 weeks, a considerable change in mineralized tissue was observed in the central part of the defects and very close to the borders contiguous to the basal bone (Figure 6A). A representative clinical view of the defect area, which decreased 8 weeks after the placement of the grafts, is depicted in Figure 6B. In addition, remnants composed of chitosan fibers and NHAPs surrounding osteoid material were observed, reducing the residual material, similar to the results obtained at 6 weeks in the same animal model but with the demineralized bone matrix [27].

A significant amount of laminar bone is present at the edges of the defects in the central part of the calvaria after implantation of colloidal AgNP-Q chitosan dispersion at 4 weeks. On the other hand, near-complete defect closure was carried out, and the thickness of the new bone formed was similar to that of the original bone (Figure 6B). At 8 weeks, the mineralized material was slightly higher than at 4 weeks. However, the bony bridge between the newly formed bone and the basal bone was only present in some small areas and was not entirely uniform. Moreover, a homogeneous distribution of the bone tissue was observed, with little or no residual material being distinguished by the presence of new mineralized material with osteoblasts and osteocytes within its matrix (Figure 6C).

### 3.8. Radiographic Evaluation 

Once the calvarias were dissected, a conventional periapical X-ray was taken and digitized for processing with the ImageJ imaging program. The evaluation of the 8 mm defects grafted with colloidal NHAP-Q chitosan dispersion at 4 weeks of evolution showed an average decrease in its initial area of 37.5 ± 4.0%, while a 75.1 ± 12.1% reduction was observed at 8 weeks. On the other hand, a decrease of 67.5 ± 5.7% was observed at 8 weeks, which was more significant (79.3 ± 11%) at 8 weeks of evolution for defects implanted with colloidal AgNP-Q chitosan dispersion (Table 2). 

### 3.9. Scanning Electron Microscopy Evaluation of the Calvarias

**Figure 7:**
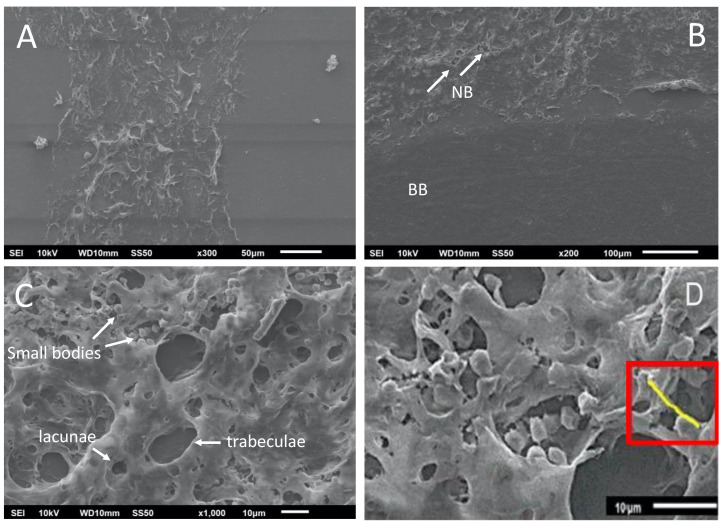
Scanning electron microscopy images of the calvaria at 8 weeks post-treatment. (**A**) De novo-formed bone; (**B**) de novo-formed bone showing bone trabeculae formation (white arrows) and gaps. NB, de novo-formed bone; BB, basal bone. (**C**) Small cell bodies correspond to osteoblasts and osteocytes. (**D**) Osteoid deposit after mineralization (red rectangle). Scale bars are indicated at the bottom of each image.

The interfaces between the new bone formed, the basal bone, and the center of the graft were analyzed (Figure 7A), including the image of the de novo-formed bone with the presence of bone trabeculae formation and gaps (Figure 7B). The interconnection points with the basal bone support the integration of the de novo-formed bone. In a close-up analysis, small cell bodies were observed, corresponding to osteoblasts and osteocytes in the bone trabeculae and the lagoons, respectively (Figure 7C). These osteoblasts are essential for depositing new non-mineralized tissue (osteoid) that will later be mineralized (Figure 7D), where structures called burr-like projections connect the osteoblasts. These structures are evidence of the deposition of mineralizable substances [28].

### 3.10. Blood Analysis

Analysis of the blood of animals treated with the colloidal chitosan dispersions versus the untreated control showed that only AST was significantly different. The rest of the blood parameters showed no significant difference, suggesting that the use of the colloidal chitosan dispersions is safe for different organs (liver and kidney), as no significant differences in ALT, urea, or creatinine were measured (Table S1). 

### 3.11. Distribution of Silver in Organs

Our study analyzed the Ag content in the heart, liver, kidneys, spleen, and skin. The liver and kidneys were assessed due to their function as excretion systems in the body. In contrast, the spleen was analyzed because it functions as a lymphoid organ and recycles the erythrocytes. In the case of the heart and skin, they were interpreted as potential places for Ag deposition through blood circulation or direct contact with the colloidal chitosan dispersion. Histological analysis of these organs showed no changes in the ultrastructure and morphology of the tissues (Figure S4). 

The results showed a low accumulation of Ag in the tested tissues (Table S2). Although a similar study showed higher accumulation in the kidneys and liver [29], this difference could be related to the size of the nanoparticles used (36 nm versus 13 nm in our study).

## 4. Discussion

It is widely known that colloidal chitosan dispersions have a wide variety of characteristics that make them unique and ideal for use in biomedicine, such as oxygen permeability, the presence of metabolites, and good mechanical properties that mimic tissues [30]. This is especially relevant when transplants are needed for bone regeneration. However, autografts can have many complications and be hard to source for areas such as the oral cavity [31].

Our study developed colloidal chitosan dispersions combined with AgNP-Q and NHAP-Q with high efficacy in bone healing. This effect is unsurprising as previous studies have shown that combined chitosan and HA composites have promising properties in bone regeneration. These include controllable porosity, water retention, tensile strength, and the ability to have pre-osteoblast cells (MC3T3-E1) attach and proliferate, creating a suitable environment for cell crawling and growth, without an impact on cell morphology and viability [32,33]. In addition, it has been shown that a chitosan-HA membrane would be more effective than chitosan alone for bone formation in the early stages [34].

A cytotoxic effect was measured in both cell lines in an in vitro study using osteoblast and osteoclast cell lines exposed to 50 nm AgNPs (surface area: 11.54 m^2^/g). The cytotoxic effect was based on the size- and area-dependent release of Ag^+^ from the NPs, with 3 µm microparticles (surface area: 0.19 m^2^/g) displaying weak cytotoxic effects and 30 µm microparticles (surface area: 0.019 m^2^/g) having no inhibitory effects at all. Osteoblasts seemed to be more sensitive, with viability and differentiation significantly decreased at AgNP treatments of 128 µg/mL and 64 µg/mL, respectively, while osteoclasts experienced significant decreases at 256 µg/mL and 128 µg/mL, respectively [35]. However, this study also found that the antibacterial effects on *S. epidermidis* were only effective at Ag^+^ concentrations of 2–4× the cytotoxic levels. This underlines the importance of customizing NP size to optimize toxicity and antibacterial activity. 

Interestingly, mesenchymal stem cells (MSCs) were exposed to AgNPs in the stage of osteoblast differentiation. The results showed that AgNPs induced the expression of the gene *CBFA1* responsible for the osteoblast differentiation [36]. Although we did not test the sole effect of AgNPs in osteoblasts, our results showed osteoblast proliferation using colloidal chitosan dispersions containing AgNPs, suggesting that the presence of the colloidal chitosan dispersion component did not affect the growth of osteoblasts. 

Regarding the biocompatibility of our colloidal chitosan dispersions, our results indicated that they are biocompatible as a faster reparation of the defect in the in vivo study (protocol B) was observed. In addition, it is well known that AgNPs have a potent antibacterial activity not present in the NHAPs. On the other hand, using AgNPs alone raises concerns about their distribution in organs when used in an in vivo protocol. Those are the main reasons why we chose to perform more studies with AgNPs than NHAPs.

Other studies reported similar results of biocompatibility compared to our colloidal chitosan dispersions. For example, good compatibilities were obtained when natural rubber latex-AgNP composites were applied to regenerate bone defects [37]. Similar results were obtained when AgNPs were added to a gelatin/alginate scaffold used for skull defect treatment [38]. Another composite prepared with polyurethane and tannic acid supplemented with AgNP and NHAP reported similar results compared to our study for the calvaria regeneration [39]. 

One study attempted to use Ag and its innate antimicrobial abilities to combat implant infection, creating an Ag coordination polymer network presented as a nanostructured coating on metallic implant substrates. These networks showed dose-dependent antibacterial effects on *S. epidermidis* and *S. aureus* in agar inhibition assays and prevent of *S. epidermidis* implant infection in murine models. Some studies have attributed the toxicity of AgNPs to a wide variety of mechanisms, including the release of ions from NPs, oxidative stress, protein or DNA binding, and the generation of reactive oxygen species [40]. In addition, the mechanism of toxicity depends on the characteristics of NPs, such as their surface area, size, shape, capping agent, surface charge, particle purity, structural distortion, and the bioavailability of the individual particles [40]. 

AgNPs have also been found to promote osteogenic activity, induce actin polymerization, activate RhoA (which enhances actin cytoskeletal tension and induces osteogenesis), and increase the expression of multiple genes, including *RUNX2*, *ALP*, *BMP-2*, *COL1A1*, *OCN*, and *OPN* when compared to AgNO_3_ exposure. Low AgNP levels that were not cytotoxic to urine-derived mesenchymal stem cells (≤4 µg/mL) still increased ALP activity and matrix mineralization [41]. Another study reported the in vitro effect of AgNPs on cell calcification and mineralization by bone cells. A remarkable cell mineralization enhancement coincided with AgNP exposure, followed by HANP exposure. In addition, AgNPs were found to alter the microRNA expression of transcriptional factors associated with bone formation and lead to miRNA regulation of several gene-target BMPs not found in the controls. Cytotoxicity studies performed with the NP concentrations used in the experiments did not indicate any onset of cytotoxic effects [41].

Furthermore, the addition of Ag into scaffolds has been performed in many studies. An in vitro study with human osteoblast-like cells (MG63) showed cytocompatibility with the treatment of a membrane made from a slurry of Ag ion-substituted nano-HA/TiO_2_ NPs and polyamide 66 (Ag-nHA/TiO_2_/PA66) causing no significant viability change after up to 7 days of culture time. Another study found that adding AgNPs to a gelatin alginate scaffold increased pore size, porosity, water absorption, and degradation rates. Moreover, the 200 µM treatment was the most effective, showing the most de novo-formed bone in rabbit skull defects [38]. Contrarily, another study found that adding Ag to a chitosan nHA scaffold decreased the biodegradation rate. This could enable scaffold availability during an extended period of bone growth. In addition, the composite was antibacterial against *S. aureus* and *E. coli* while remaining non-toxic to rat primary calvarial osteoblasts and a human osteosarcoma cell line [42]. As compounds in each scaffold differ, it is complicated to compare results, as any discrepancies can be due to the properties of selected materials. However, it remains clear that Ag addition to composites contributes as an antibacterial component and aids in refining parameters. 

Simultaneously, it is important to balance the unique properties with toxicity to ensure cytocompatibility. In vivo studies showed significant changes in rats’ ALP and cholesterol levels upon AgNP exposure, suggesting that exposure to more than 300 mg of AgNPs might result in mild liver damage [43,44]. Another study reported a toxicological evaluation of male guinea pigs exposed to three concentrations of AgNPs (100, 1000, and 10,000 ppm) in a sub-chronic model for 13 weeks. A close correlation between the dermal exposure and tissue levels of AgNPs was found (*p* < 0.05), and tissue uptake of AgNPs happened in a dose-dependent manner with the following ranking: kidney > muscle > bone > skin > liver > heart > spleen. In the same study, histopathological analyses showed severe proximal convoluted tubule and distal convoluted tubule degeneration in the kidneys of the middle- and high-dose animals. Separate lines and marrow space narrowing were determined as two significant signs of bone toxicities observed in the three different dose levels of AgNPs. Moreover, an increased dermal dose of AgNPs caused cardiocyte deformity, congestion, and inflammation [45]. In contrast, the concentration of Ag in our colloidal chitosan dispersions was 1 mg/mL. No toxicity or histological changes in the tibiae and calvarias were observed at this concentration. On the other hand, when the control skin was compared with the treated skin (Figure S2), a minor inflammatory infiltrate was observed in the treated skin, with high fibroblastic activity and collagen deposits. These activities suggest an accumulation of the AgNPs on the treated site. However, this is a weak response to the colloidal chitosan dispersion but not to a foreign implanted body that would produce giant cells and it did not alter the tissue repair process [46]. 

Our results showed that colloidal NHAP-Q chitosan dispersion regenerated the bone to 75% at 8 weeks. This result was superior to some other studies; for example, a bone formation of 67%, 57%, 50%, 19%, and 11% was measured when the macroporous biphasic calcium phosphate, Bio-Oss (Geistlich, Princeton, NJ, USA), demineralized freeze-dried bone, allograft, Stypro, and empty control were evaluated, respectively [47]. A study using chitosan combined with an absorbable collagen sponge reported a defect closure of 2.83 ± 1.06% and a new bone area of 4.84 ± 0.88% at 8 weeks. Interestingly, there was also a significant decrease in bone density at 8 weeks compared with controls, which the authors attributed to the remainder of residual materials, fatty marrow, and fibrovascular tissue [10]. However, one study reported the complete healing of 5 mm defects in rat calvarias after 8 weeks of implantation using an Ag-nHa/TiO_2_/PA66 membrane [48]. Again, because the tested substrates are different in experiments, it is hard to compare them with our results directly. Additionally, many studies reported a qualitative observation rather than a percentage. For example, one study (using a membrane formulated with HA and chitosan) reported improved local bone tissue growth in an 8 mm rat calvarial critical-size defect at 2 and 8 weeks [34]. The efficacy was significantly higher than the control membrane, which did not contain HA. Another study using colloidal chitosan dispersions made with hyaluronate and MegaGen synthetic bone reported colloidal chitosan dispersion resorption and partial substitution to the lamellar bone after 8 weeks of implantation [49].

## 5. Conclusions

The biological implication of our study is that our colloidal chitosan dispersion mimics an extracellular matrix, which is not similar to a conventional rigid graft. Moreover, our porous chitosan colloidal dispersions allow cellular dynamics in any direction where cellular integration is facilitated [30], showed biocompatibility, and promoted osteoregeneration. Moreover, we demonstrated significant antibacterial activity against clinical multidrug-resistant bacteria, and in vivo studies confirmed significant healing of CSDs. This study showed a potential for future use of our nanocomposites in biomedical applications. The advantage of the proposed use of the nanocomposites developed in this study is that in future applications the concentrations of the AgNPs, HANP, and chitosan could be adapted to a specific application for better efficacy. For example, the concentration of the AgNPs could be increased according to the presence of multidrug-resistant bacteria.

## Figures and Tables

**Table 1:** Antibacterial activity of colloidal chitosan dispersions against multidrug-resistant bacteria expressed as MIC (μg/mL).

Bacterial Strain	Colloidal AgNP-Q Chitosan Dispersion NPs	Colloidal Chitosan Dispersions Control
*Acinetobacter baumannii*	12.5	R
*Staphylococcus epidermidis* 6	7.5	R
*Staphylococcus epidermidis* 710	12.5	R
*Staphylococcus aureus* 700	20	R

R = resistant.

**Table 2:** Mean bone formation (*n* = 5) expressed as % ± SD.

Weeks *	Colloidal NHAP-Q Chitosan Dispersion	Colloidal AgNP-Q Chitosan Dispersion
4	37.5 ± 4.0	67.5 ± 5.7
8	75.1 ± 12	79.3 ± 11

* Time after the procedure.

## Data Availability

The data that support the findings of this study are available from the corresponding author upon reasonable request.

## Supplementary Materials

The following supporting information can be downloaded at: https://www.mdpi.com/article/10.3390/nano13020321/s1, Figure S1: Surgical sites in Protocol A (Biocompatibility); Figure S2: Clinical observation of the defects after 4 weeks post-treatment; Figure S3: Characterization of the NPs used in this study by zeta potential; Figure S4: Representative images of organ histology; Table S1: Blood parameters were measured in animals of animals at 8 weeks expressed as a mean of *n* = 5; Table S2: Tissue levels of Ag expressed as mg/g wet tissue, *n* = 5.
