# Supplementary material for: Osteoregeneration of Critical-Size Defects Using Hydroxyapatite–Chitosan and Silver–Chitosan Nanocomposites
Source: Nanomaterials (Basel). 2023 Jan 12;13(2):321. doi: 10.3390/nano13020321 (PMC9861689; doi:10.3390/nano13020321)
Supplement: Supplementary file 1 [file nanomaterials-13-00321-s001.zip › nanomaterials-2142882-supplementary.pdf]

Supplemental Information

Osteoregeneration of Critical-Size Defects Using Hydroxyapatite–Chitosan and Silver–Chitosan Nanocomposites

Miguel A. Casillas-Santana [1], Yael N. Slavin [2], Peng Zhang [2], Nereyda Niño-Martínez [3], Horacio Bach [2,*] and Gabriel A. Martínez-Castañón [1,*]

[1] Laboratorio de Nanobiomateriales, Facultad de Estomatología, Universidad Autónoma de San Luis Potosí, San Luis Potosí 78290, Mexico;
[2] Division of Infectious Diseases, Faculty of Medicine, University of British Columbia, Vancouver, BC V6G3Z6, Canada
[3] Facultad de Ciencias, Universidad Autónoma de San Luis Potosi, San Luis Potosí 78295, Mexico
* Correspondence: hbach@mail.ubc.ca (H.B.); mtzcastanon@fciencias.uaslp.mx (G.A.M.-C.)

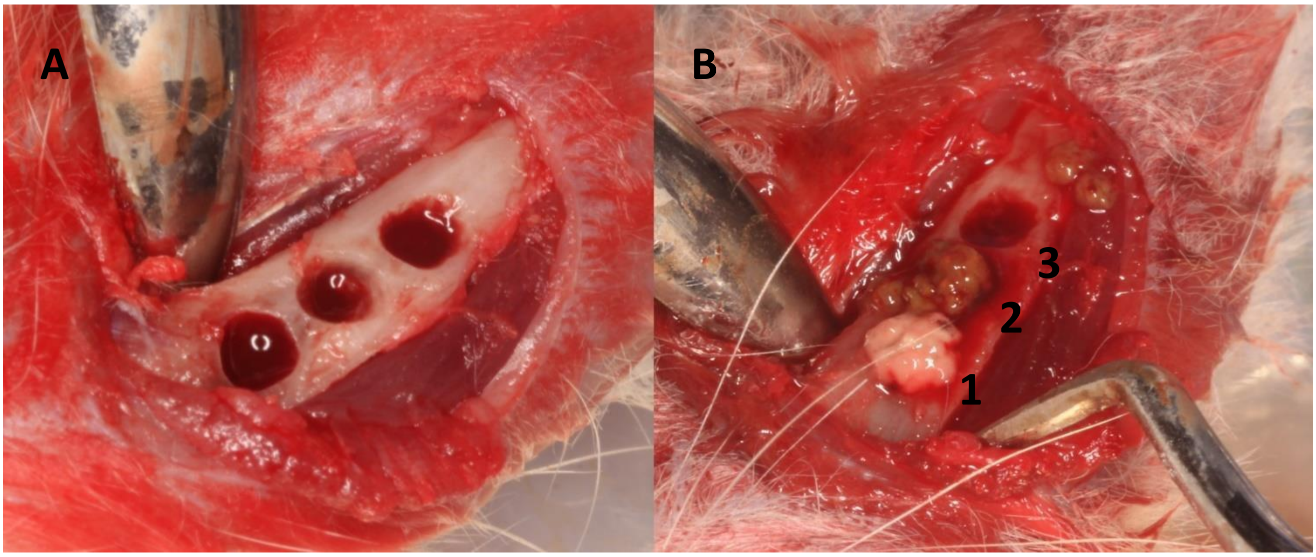

Figure S1. Surgical sites in Protocol A (Biocompatibility). (A) The bone was drilled, and three defects were performed. (B) The defects were filled with (1) NHAP-Q, (2) AgNP-Q, and (3) Blood cloth as a control. .

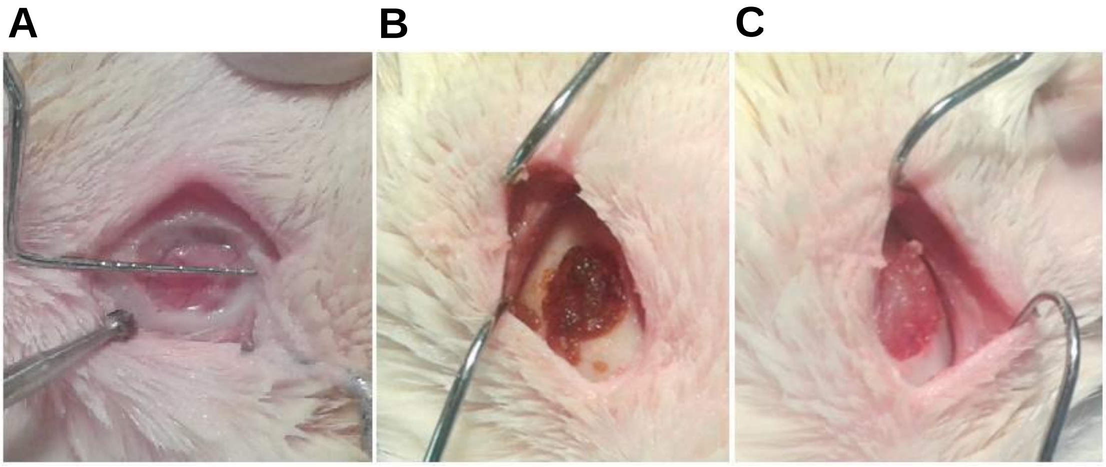

Figure S2. Clinical observation of the defects after 4 weeks post-treatment. (A) Control, (B) AgNP-Q, (C) NHAP-Q.

A

		Mean (mV)	Area (%)	Width (mV)
**Zeta Potential (mV):** -48,4	**Peak 1:**	-48,4	100,0	6,96
**Zeta Deviation (mV):** 6,96	**Peak 2:**	0,00	0,0	0,00
**Conductivity (S/m):** 0,00207	**Peak 3:**	0,00	0,0	0,00

Result quality Good

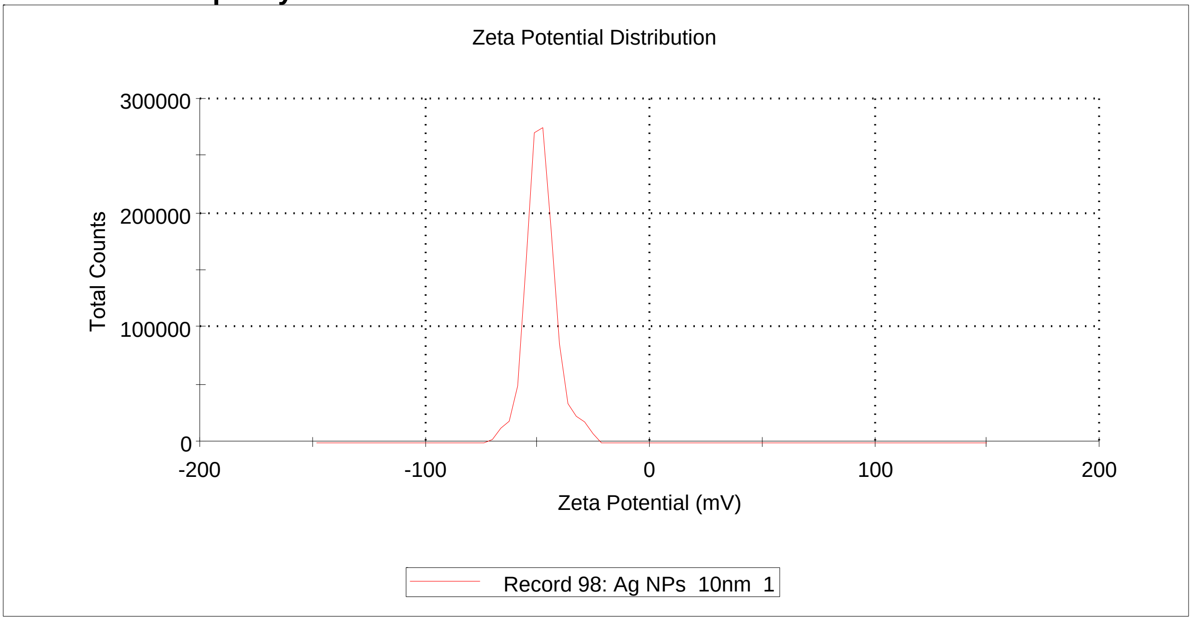

B

			Mean (mV)	Area (%)	Width (mV)
**Zeta Potential (mV):**	-26,8	**Peak 1:**	-26,8	100,0	8,42
**Zeta Deviation (mV):**	8,42	**Peak 2:**	0,00		0,00
**Conductivity (S/m):**	0,00199	**Peak 3:**	0,00		0,00
**Result quality** Good					

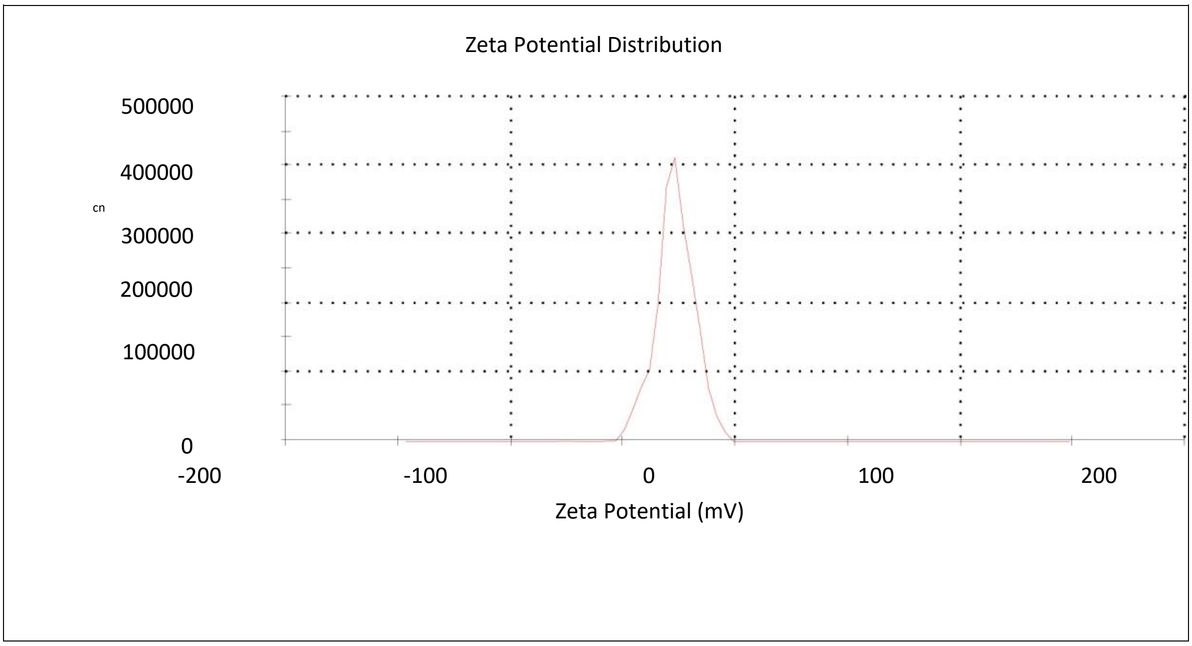

Figure S3. Characterization of the NPs used in this study by zeta potential. (A) AgNPs (B) NHAPs.

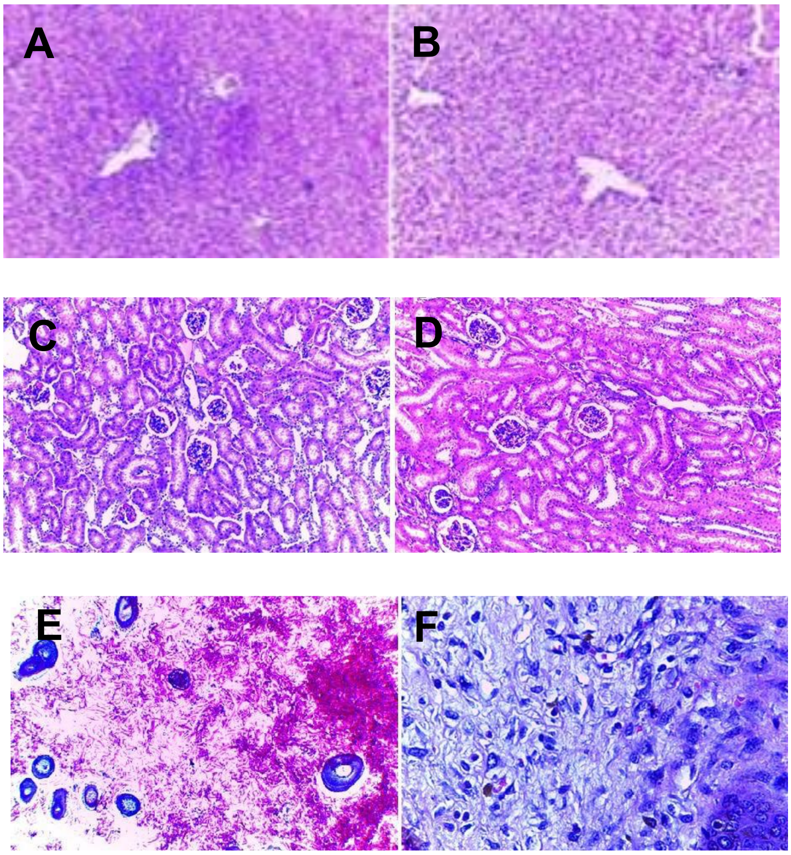

Figure S4. Representative images of organ histology. (A) Liver control, (B) Liver after treatment, (C) Kidney control, (D) Kidney after treatment, (E) Skin control, (F) Skin after treatment.

Table S1. Blood parameters were measured in animals of animals at 8 weeks expressed as a mean of n=5.

	ALT	AST	U	Cr	WBC	LYM	GRA	RBC	Hg	PLT	HCT	MCV	MCH
Treated	103	171	35	0.5	8.88	4.62	1	4.6	14	491	36.9	79.5	30.3
Control	92	62	39	0.4	7.32	3.94	1.06	4.9	13.7	434	36.3	74.1	28.1

ALT= alanine aminotransferase (U/L), AST= aspartate aminotransferase (U/L), U= urea (mg/dL), Cr= creatinine (mg/dL), WBC= white blood cells (10^6/mL), LYM= lymphocytes (10^6/mL), GRA= neutrophils (10^6/mL), RBC= red blood cells (10^6/mL), Hg= hemoglobin (g/dL), PLT= platelets (10^6/mL), HCT= hematocrit (%), MCV= mean corpuscular volume (fL), MCH= mean corpuscular hemoglobin (pg).

Table S2. Tissue levels of Ag expressed as µg/g wet tissue, n=5. .

Heart	Skin	Kidney	Liver	Spleen	Size (nm)	Time (days)	Reference
1.38	1.83	0.95	0.93	4.62	13	56	This study
ND	ND	25.5	12	ND	36	56	[1]
ND	ND	2.5	1	ND	14	28	[2]

ND, not determined.

References

1. Espinosa-Cristobal, L.F.; Martinez-Castañon, G.A.; Loyola-Rodriguez, J.P.; Patiño-Marin, N.; Reyes-Macías, J.F.; Vargas-Morales, J.M.; Ruiz, F. Toxicity, Distribution, and Accumulation of Silver Nanoparticles in Wistar Rats. *J. Nanopart. Res.* **2013**, *15*, 1702. https://doi.org/10.1007/s11051-013-1702-6.
2. Loeschner, K.; Hadrup, N.; Qvortrup, K.; Larsen, A.; Gao, X.; Vogel, U.; Mortensen, A.; Lam, H.R.; Larsen, E.H. Distribution of Silver in Rats Following 28 Days of Repeated Oral Exposure to Silver Nanoparticles or Silver Acetate. *Part. Fibre Toxicol.* **2011**, *8*, 18. https://doi.org/10.1186/1743-8977-8-18.
